# Unusual course of outflow tract tachycardia and cardiac decompensation in childhood: a case report and review of the literature

**DOI:** 10.1007/s00399-025-01090-w

**Published:** 2025-07-25

**Authors:** Antonia Weymann, Julia Köbe, Julia Stegger, Julian Wolfes, Lars Eckardt, Matthias Sigler

**Affiliations:** 1 Klinik für Kinder- und Jugendmedizin, Pädiatrische Kardiologie, Universitätsklinikum Münster, Alber-Schweitzer Campus 1, 48149 Münster, Germany; 2 Klinik für Kardiologie II—Rhythmologie, Universitätsklinikum Münster, Münster, Germany

**Keywords:** Idiopathic ventricular tachycardia, Right ventriculra outflow tract tachycardia in children, Radiofrequency ablation in children, Laminopathy, Left ventricular dysfunction, Idiopathische ventrikuläre Tachykardie, Rechtsventrikuläre Ausflusstrakttachykardie im Kindesalter, Radiofrequenzablation im Kindesalter, Laminopathie, Linksventrikuläre Dysfunktion

## Abstract

Outflow tract tachycardia (OT) is the most common form of idiopathic ventricular tachycardia (VT) in children. It is usually an incidental finding and presents only with mild symptoms. If affected patients have impaired left ventricular (LV) function, recovery usually progresses quickly after termination of the VT. To the best of the authors’ knowledge, this is the first description of a 9-year-old female patient who presented with incessant right ventricular OT and LV dysfunction that was initially assumed to be tachycardiomyopathy and later diagnosed to be related to underlying laminopathy. The VT was successfully treated by radiofrequency ablation, and electroanatomical mapping demonstrated no signs of endocardial scarring. The VT had most likely been present for a prolonged period since the patient and initially presented with only mild symptoms of cardiac congestion despite severely impaired LV function. It seems probable that the laminopathy somehow influenced the unusual course of impaired LV function in this patient, who had either idiopathic VT combined with incidentally proven laminopathy or, less likely, outflow tract VT related to the laminopathy mimicking idiopathic VT.

## Abstract (de)

Ausflusstrakttachykardien sind die häufigste Form der idiopathischen ventrikulären Tachykardie (VT) im Kindesalter. Meist handelt es sich um einen Zufallsbefund, verbunden mit nur leichten Symptomen. Haben die betroffenen Patienten eine gestörte linksventrikuläre (LV) Funktion, kommt es nach Beendigung der VT gewöhnlich zu einer schnellen Erholung. Nach unserem Wissen ist dies die erste Beschreibung einer 9‑jährigen Patientin mit permanenter rechtsventrikulärer Ausflusstrakttachykardie und LV-Dysfunktion, die zunächst als Tachykardiomyopathie gedeutet und später mit einer zugrunde liegenden Laminopathie in Zusammenhang gebracht wurde. Die VT wurde unter Anwendung der Radiofrequenzablation erfolgreich behandelt, und eine elektroanatomische Kartierung ergab keine Anzeichen für endokardiale Narbenbildung. Die VT hatte wahrscheinlich schon längere Zeit bestanden, da die Patientin anfänglich trotz sehr eingeschränkter LV-Funktion nur leichte Symptome einer kardialen Stauung aufwies. Es erscheint wahrscheinlich, dass die Laminopathie auf irgendeine Weise den ungewöhnlichen Verlauf der eingeschränkten LV-Funktion im beschriebenen Fall beeinflusst hat, wobei die Patientin entweder eine idiopathische VT mit zufällig diagnostizierter Laminopathie oder – weniger wahrscheinlich – eine Ausflusstrakt-VT im Zusammenhang mit der Laminopathie unter Vortäuschung einer idiopathischen VT hatte.

## Introduction

Ventricular outflow tract tachycardias are mainly idiopathic ventricular tachycardia (VT) predominately originating from the right ventricular (RV) outflow tract (OT) (RVOT). They typically occur in young adults but may also be seen in children. They represent the most common form of VT in patients without evidence of structural heart disease and are characterized by a focal monomorphic tachycardia characterized by left bundle branch block (LBBB) morphology and an inferior axis [1, 13]. OT tachycardia carries a favorable prognosis despite some reported cases of sudden cardiac death (SCD). Patients may be asymptomatic or present with palpitations or dizziness, and syncope may rarely occur during VT [11].

## Case report

**Fig. 1:**
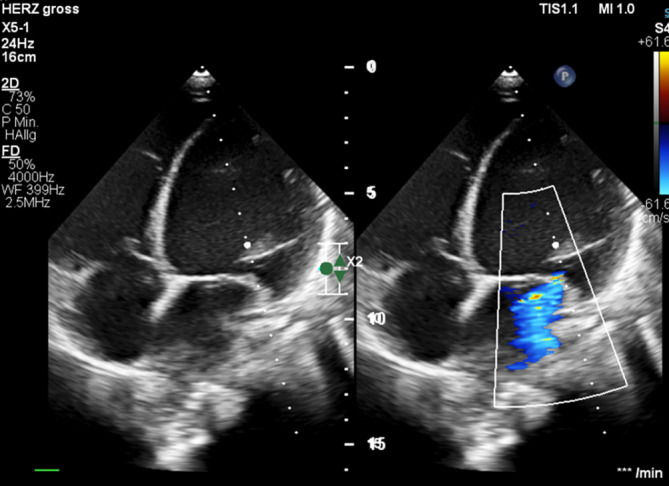
Echocardiography on admission. Note left ventricular dilatation and mitral valve insufficiency

A 9-year-old girl presented with slight deterioration in her general condition over the preceding 2 weeks. She was referred by her pediatrician due to bilateral eyelid edema, hepatomegaly on palpation and cough persisting for 2 days. Chest X‑ray revealed cardiomegaly. Echocardiography showed left ventricular (LV) dilation (LV end-diastolic diameter 57 mm) with second-degree mitral valve insufficiency (Fig. 1). LV contractility was severely impaired (fractional shortening [FS] 12%, LV dP/dt 630 mm Hg/s). Correspondingly, laboratory tests revealed an elevated NT-proBNP level of 12,006 pg/ml (normal range 8–178 pg/ml). Relative to her severely impaired ventricular function, the patient presented with a surprisingly stable general condition with only mild symptoms. The family history was unremarkably. Cardiac magnetic resonance imaging (MRI) confirmed echocardiographic findings, demonstrating an end-diastolic LV volume of 203 ml (188 ml/m^2^; reference range: 60–70 ml/m^2^) and an LV ejection fraction (LV-EF) of 16%. The right ventricle (RV) was also dilated, with an end-diastolic volume of 120 ml (112 ml/m^2^; reference range: 62–72 ml/m^2^) and a reduced RV-EF of 35%. There was no evidence for late gadolinium enhancement (LGE).

**Fig. 2:**
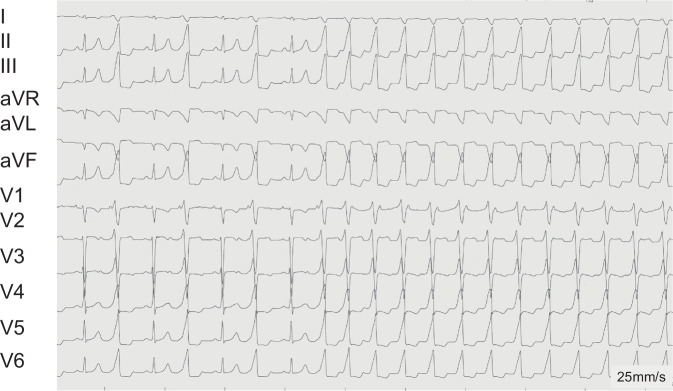
A 12-lead ECG of a 9-year-old girl with almost incessant right ventricular outflow tract (RVOT) ventricular tachycardia (VT). Note the initial ventricular bigeminus and the transition to a sustained RVOT VT

**Fig. 3:**
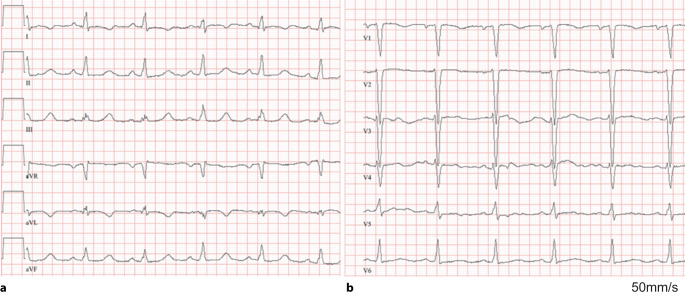
A 12-lead ECG of a 9-year-old girl with almost incessant right ventricular outflow tract (RVOT) ventricular tachycardia (VT) (see Fig. 2) during a short VT pause

During monitoring, almost incessant wide QRS complex tachycardias with a rate of 130–140 bpm were observed. The ECG (Fig. 2) exhibited characteristics of an RVOT tachycardia, including an inferior axis and an LBBB pattern with R–S transition in V_3_–V_4_. Sinus rhythm without pre-excitation and normal atrioventricular (AV) conduction (PR interval of 100 ms) was observed during intermittent short periods with spontaneous termination of the tachycardia (Fig. 3).

**Fig. 4:**
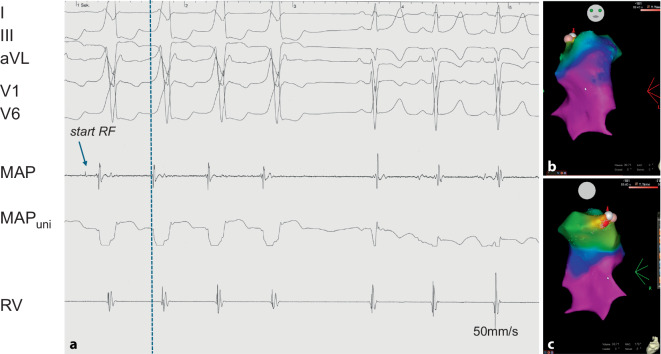
Intracardiac recordings in a 9-year-old girl with almost incessant right ventricular outflow tract (RVOT) ventricular tachycardia. The signals of the ablation catheter depict the bipolar (MAP) and unipolar (MAPuni) recordings at the successful ablation site. **b,** **c** An electroanatomical CARTO map with an anterior (**b**) and posterior (**c**) view of the RVOT with the successful ablation site (red pin)

Due to the severely impaired cardiac function, pharmacological circulatory support with milrinone was initiated. Assuming a tachycardiomyopathy, an electrophysiologic study was scheduled and conducted under sedation with dexmedetomidine. The study was performed using 3D electroanatomical mapping (CARTO, Johnson & Johnson Inc., Irvine, CA, USA). During tachycardia, the earliest activation was identified in the distal septal region of the RVOT, just below the pulmonary valve (Fig. 4). The tachycardia almost immediately terminated after starting radiofrequency ablation at the exit site. It was no longer inducible thereafter. In line with the assumed idiopathic VT, electroanatomical voltage mapping of the RV revealed no significant substrate abnormalities. In the subsequent course, neither tachycardia nor other arrhythmias recurred. As part of the anti-congestive medical regimen, the patient was being treated with lisinopril, bisoprolol, hydrochlorothiazide, and spironolactone. However, LV function improved only gradually. At 4 weeks post-ablation, the FS was still 16%, and LV dP/dt was 730 mm Hg/s, but the NT-proBNP level had decreased to 2171 pg/ml. Visually, there was a slight but unambiguous improvement of the wall motion pattern. As LV function improved only slightly, genetic testing was performed, revealing a most likely pathogenic lamin A/C (*LMNA*) mutation and leading to the diagnosis of a genetically determined dilated cardiomyopathy.

## Discussion

To the best of the authors’ knowledge, this is the first case of an assumed almost incessant idiopathic outflow tract tachycardia in a child with *LMNA* cardiomyopathy. Due to the normal endocardial voltage map and the lack of LGE on cardiac MRI, an accidental association is likely. However, one may speculate that the “idiopathic” RVOT arrhythmia may have triggered the cardiac decompensation in this 9‑year-old girl with *LMNA* cardiomyopathy, and it is also not possible to exclude a potential causal association between the RVOT tachycardia with the *LMNA* cardiomyopathy. However, a directly linked association between *LMNA* cardiomyopathy and OT tachycardia has not been described as yet.

Pathogenic mutations are identified in 25–55% of patients with dilated cardiomyopathy (DCM) [10]. Mutations in genes such as *LMNA, PLN, RBM20*, and *FLNC* are associated with an increased risk of ventricular arrhythmias (VA) and SCD. *LMNA* mutations represent 5–10% of all DCM patients [10, 16]. Independent of LV-EF, carriers of desmosomal and *LMNA* mutations experience the highest rates of VA/SCD [6]. An inherited DCM is also more likely in patients who present at young age or with signs suspicious for a specific etiology (e.g., prolonged AV conduction for *LMNA*), but the presented manifestation before the age of 10 years seems particularly unusual. Most children are detected by cascade screening of affected adults but not as index patients.

Regarding SCD, stratification is particular challenging in childhood. There are no evidence-based recommendations for primary prevention in children with *LMNA* cardiomyopathy. A risk calculator has recently been developed (https://lmna-risk-vta.fr) in an adult population and is probably not transferable to children [14]. Thus, primary prevention with an implantable cardioverter-defibrillator (ICD) can only be recommended on an individual basis. Close follow-up with regular evaluation of LV-EF, ECG and HOLTER recordings as well as cardiac MRI (LGE) may guide therapy in one direction or the other.

Our patient presented with an electrophysiologically typical RVOT tachycardia, which was successfully ablated. OT tachycardias are the most common form of VT in children [7]. One of the largest VT studies in pediatric patients was reported by Pfammatter et al. [12] and concluded that most of the VT in children originate from the RVOT (70%). Table 1 summarizes the largest series of catheter ablation for idiopathic VT in children.

**Table 1:** Overview of observational studies on catheter ablation of ventricular tachycardias in children with more than 20 patients

	Dalili [3]	Chiu [2]	Li [11]	Kilic [8]	Fukuhara [5]	Harris [7]	Pfammatter [12]
*No. of patients*	102	57	92	22	46	48	98
*Age*	9 mo–16 y	5.3–14.7 y	3.6–18 y	1 mo–16 y	4–19 y	1.5 mo–17 y	1.5 mo–15 y^3^
*Asymptomatic*	0 (0%)	53 (93%)	63 (68%)	17 (77%)	34 (74%)	22 (46%)	36 (37%)
*Syncope*	55 (54%)	4 (7%)	na	1 (4%)	8 (17%)	7 (15%)	4 (4%)
*Palpitations*	61 (60%)	37 (65%)	na	15 (68%)	17 (37%)	na	12 (12%)
*Compromised cardiac function*	43 (42%)^1^	8 (14%)	15 (16%)	na	na	1 (2%)	11 (11%)
*RVOT VT*	35 (34%)	na	69 (75%)	na	21 (46%)	48 (100%)	5 (5%)^4^
*Radiofrequency ablation (RFA)*	116^2^	40 (75%)	92 (100%)	12 (54%)	32 (70%)	6 (13%)	9 (9%)
*Successful RFA*	99 (85%)	36 (90%)	84 (91%)	9 (75%)	22 (69%)	4 (67%)	7 (78%)
*Mapping*	3D (*n* = 30), 2D	na	3D (*n* = 51) 2D (*n* = 42)	Non-contact mapping	3D	na	Pace mapping
*3D mapping system*	NavX; CARTO	na	CARTO	na	CARTO	na	na

^1^ 5 Patients with severe cardiac impairment

^2^ 116 Procedures in 102 patients

^3^ Ablation performed at the age of 3–13 years

^4^ VT origin was examined in only nine patients

*na* Not available

Using 3D electroanatomical mapping, ablation success rates have increased over recent years with very low complication rates [4]. Thus, first-line catheter ablation of RVOT tachycardia has been recommended for adults in recent European Society of Cardiology (ESC) guidelines [16] and other international recommendations [9]. These recommendations should be transferred to children on an individual basis depending on symptoms, severity, prevalence of VT and LV function. Additionally, patient age and body size are critical factors influencing the timing and indication of catheter ablation, as they directly impact both the procedural risk—particularly with regard to potential injury to the specialized conduction system and coronary arteries—and the technical feasibility, including catheter selection and vascular access. In our case, it is of note that the patient exhibited significant LV dysfunction, albeit accompanied by surprisingly mild clinical symptoms. This was objectively demonstrated in cardiopulmonary exercise testing (after tachycardia termination) with a maximal oxygen uptake of 32 ml/kg/min, which was within the normal range. This finding suggests a very slow progression of ventricular dysfunction, allowing the body to adapt to the reduced cardiac output. The initially assumed potential cause of the impaired ventricular function in the authors’ patient was the almost incessant RVOT tachycardia itself, i.e., tachycardiomyopathy. However, RVOT tachycardia may rarely lead to such a degree of ventricular dysfunction [17] and is normally associated with a high potential of complete recovery after VT ablation [15].

In the present case, the presence of an *LMNA* mutation combined with the co-occurring RVOT tachycardia most likely exacerbated the severity of ventricular dysfunction due to a pre-existing underlying genetic cardiomyopathy. It remains to be seen whether the patient will experience further improvement in ventricular function over the coming months. Given the progressive nature of laminopathies and the potential future necessity for electrical device therapy and possibly heart transplantation, the patient will undergo very close follow-up. Anticongestive therapy including lisinopril, bisoprolol, hydrochlorthiazide and spironolactone are continued.

## Data Availability

Not applicable

## Declarations

Informed consent was obtained from the patient and her parents.
